# Evaluation of an Automated Rapid Diagnostic Assay for Detection of Gram-Negative Bacteria and Their Drug-Resistance Genes in Positive Blood Cultures

**DOI:** 10.1371/journal.pone.0094064

**Published:** 2014-04-04

**Authors:** Masayoshi Tojo, Takahiro Fujita, Yusuke Ainoda, Maki Nagamatsu, Kayoko Hayakawa, Kazuhisa Mezaki, Aki Sakurai, Yoshinori Masui, Hirohisa Yazaki, Hiroshi Takahashi, Tohru Miyoshi-Akiyama, Kyoichi Totsuka, Teruo Kirikae, Norio Ohmagari

**Affiliations:** 1 Disease Control and Prevention Center, Research Institute, National Center for Global Health and Medicine, Tokyo, Japan; 2 Department of Infectious Diseases, Research Institute, National Center for Global Health and Medicine, Tokyo, Japan; 3 Department of Infectious Diseases, Tokyo Women's Medical University, Tokyo, Japan; 4 Department of Clinical Laboratory, National Center for Global Health and Medicine, Tokyo, Japan; 5 Department of Pulmonary Medicine, Kohnodai Hospital, National Center for Global Health and Medicine, Chiba, Japan; 6 Department of Internal Medicine, Kohnodai Hospital, National Center for Global Health and Medicine, Chiba, Japan; 7 AIDS Clinical Center, National Center for Global Health and Medicine, Tokyo, Japan; 8 East-West Diagnostics/Theranostics, LLC, San Francisco, California, United States of America; Charité, Campus Benjamin Franklin, Germany

## Abstract

We evaluated the performance of the Verigene Gram-Negative Blood Culture Nucleic Acid Test (BC-GN; Nanosphere, Northbrook, IL, USA), an automated multiplex assay for rapid identification of positive blood cultures caused by 9 Gram-negative bacteria (GNB) and for detection of 9 genes associated with β-lactam resistance. The BC-GN assay can be performed directly from positive blood cultures with 5 minutes of hands-on and 2 hours of run time per sample. A total of 397 GNB positive blood cultures were analyzed using the BC-GN assay. Of the 397 samples, 295 were simulated samples prepared by inoculating GNB into blood culture bottles, and the remaining were clinical samples from 102 patients with positive blood cultures. Aliquots of the positive blood cultures were tested by the BC-GN assay. The results of bacterial identification between the BC-GN assay and standard laboratory methods were as follows: *Acinetobacter* spp. (39 isolates for the BC-GN assay/39 for the standard methods), *Citrobacter* spp. (7/7), *Escherichia coli* (87/87), *Klebsiella oxytoca* (13/13), and *Proteus* spp. (11/11); *Enterobacter* spp. (29/30); *Klebsiella pneumoniae* (62/72); *Pseudomonas aeruginosa* (124/125); and *Serratia marcescens* (18/21); respectively. From the 102 clinical samples, 104 bacterial species were identified with the BC-GN assay, whereas 110 were identified with the standard methods. The BC-GN assay also detected all β-lactam resistance genes tested (233 genes), including 54 *bla*_CTX-M_, 119 *bla*_IMP_, 8 *bla*_KPC_, 16 *bla*_NDM_, 24 *bla*_OXA-23_, 1 *bla*_OXA-24/40_, 1 *bla*_OXA-48_, 4 *bla*_OXA-58_, and 6 *bla*_VIM_. The data shows that the BC-GN assay provides rapid detection of GNB and β-lactam resistance genes in positive blood cultures and has the potential to contributing to optimal patient management by earlier detection of major antimicrobial resistance genes.

## Introduction

Sepsis caused by drug-resistant Gram-negative bacteria (GNB) often results in serious clinical outcomes in patients [1]. Inappropriate initial antibiotic treatment occurs in one third of patients with severe sepsis due to GNB, which is associated with increased hospital mortality and length of stay [2]. In contrast, early administration of appropriate antibiotics improves survival of sepsis patients [3]. Effective antibiotic administration within the first hour of documented hypotension was related to increased survival of patients with septic shock; however, 50% of septic shock patients did not receive effective antimicrobial treatment within 6 hours of documented hypotension [4]. To improve diagnosis of causative organisms of sepsis, automated continuous-monitoring blood culture systems were developed and introduced into clinical microbiological laboratories during the 1990s [5]. These early systems and subsequent generations of automated blood culture systems remain key diagnostic tools in the diagnosis of sepsis [5]. However, after a blood culture becomes positive, conventional bacteriological procedures still require 2 to 3 days for isolation, identification, and antimicrobial susceptibility testing. In addition to the time-consuming procedures, the emergence and spread of drug-resistant GNB producing various β-lactamases, including carbapenemase and extended spectrum β-lactamases (ESBLs), has been a serious problem for treatment of sepsis [6]. Thus, there has been an unmet need for rapid and automated technology to identify bacterial species as well as detection of drug resistance genes.

Recently, several rapid molecular diagnosis assays for sepsis diagnosis have been introduced and evaluated [7]; including LightCycler SeptiFast Test [8], peptide nucleic acid fluorescence in situ hybridization (PNA-FISH) [9], and matrix-assisted laser desorption-ionization time of flight mass spectrometry (MALDI-TOF MS) [10], and a DNA-based microarray platform [Prove-it sepsis assay [11] and the Verigene Gram-Positive Blood Culture (BC-GP) assay [12]–[19]].

The Verigene Gram-Negative Blood Culture (BC-GN) Nucleic Acid Test (Nanosphere Inc., Northbrook, IL) is a sample-to-result automated microarray-based, multiplexed assay for species identification of GNB and detection of their drug resistance genes in positive blood culture bottles. The Verigene BC-GN assay is designed to directly detect species of GNB from positive blood culture bottles with 5 minutes of hands-on and 2 hours of run time per sample. Recently, the BC-GN assay has been approved by the U.S. Food and Drug Administration (FDA), and the limit of detection, sensitivity, and specificity of the assay is shown on the FDA site (http://www.fda.gov/). In this study, we describe the performance of the BC-GN assay using simulated and clinical samples of positive blood culture bottles.

## Materials and Methods

### Bacterial strains

A total of 268 stored clinical isolates at the National Center for Global Health and Medicine (NCGM) were used in the study: 23 *Acinetobacter baumannii*; 1 *Acinetobacter oleivorans*; 4 *Citrobacter freundii*; 14 *Enterobacter cloacae*; 2 *Enterobacter hormaechei*; 1 *Enterococcus faecalis*; 30 *Escherichia coli*; 6 *Klebsiella oxytoca*; 43 *Klebsiella pneumoniae*; 1 methicillin-resistant *Staphylococcus aureus* (MRSA); 1 methicillin-sensitive *Staphylococcus aureus* (MSSA); 1 methicillin-resistant *Staphylococcus epidermidis* (MRSE); 1 *Morganella morganii*; 4 *Proteus mirabilis*; 4 *Proteus vulgaris*; 116 *Pseudomonas aeruginosa*; 15 *Serratia marcescens*; and 1 *Stenotrophomonas maltophilia*. Eighteen bacterial strains were donated by Yoshikazu Ishii (Toho University, Tokyo, Japan), including 2 strains of *A. baumannii* harboring *bla*_OXA-23_; 1 *A. baumannii* harboring *bla*_OXA-58_; 1 *Acinetobacter calcoaceticus* harboring *bla*_IMP-1_; 1 *Acinetobacter nosocomialis* harboring *bla*_IMP-1_; 1 *Acinetobacter pittii* harboring *bla*_OXA-58_; 2 *E. cloacae* harboring *bla*_CTX-M-2_ and *bla*_CTX-M-9_, respectively; 6 *E. coli* harboring *bla*_CTX-M-2_, *bla*_CTX-M-3_, *bla*_CTX-M-9_, *bla*_CTX-M-14_, *bla*_CTX-M-15_, and *bla*_CTX-M-44_, respectively; 2 *K. pneumoniae* harboring *bla*_CTX-M-2_; 1 *K. pneumoniae* harboring *bla*_CTX-M-9_; and 1 *P. mirabilis* harboring *bla*_CTX-M-2_. Bacterial identification of the 18 strains were determined as described previously [20]. Fifteen bacterial strains were obtained from International Health Management Associates, Inc. (Schaumburg, IL), including 3 strains of *A. baumannii* harboring *bla*_OXA-23_; 2 *A. baumannii* harboring *bla*_OXA-58_; 1 *A. baumannii* harboring *bla*_OXA-24/40_; 1 *A. baumannii* harboring both *bla*_OXA-23_ and *bla*_OXA-58_; 2 *C. freundii* harboring *bla*_KPC-2_ and *bla*_KPC-3_, respectively; 1 *K. pneumoniae* harboring *bla*_KPC-2_; 1 *K. pneumoniae* harboring *bla*_KPC-4_; 2 *K. pneumoniae* harboring *bla*_KPC-11_; 1 *K. pneumoniae* harboring both *bla*_KPC-3_ and *bla*_CTX-M-14_; and 1 *S. marcescens* harboring *bla*_KPC-2_.

### Preparation of simulated samples

Bacterial isolates were suspended in 10-ml Falcon tubes (Becton Dickinson, Tokyo, Japan) in phosphate-buffered saline, pH 7.4. The suspension was adjusted to McFarland standard 1, followed by a dilution of 10^6^. A 0.1-ml aliquot was inoculated into 5 ml of human whole blood for blood transfusion that was scheduled for disposal (Japanese Red Cross, Kanto-Koshinetsu Block Blood Center, Tokyo, Japan). After mixing of the blood and bacterial inoculum, the sample was injected into a Bactec plus/F aerobic blood culture bottle (Becton Dickinson). A 0.1-ml aliquot from the diluted bacterial suspension was plated, and the colony-forming unit (CFU) was determined. The average CFU of the inoculum was 23.9 ± 22.6 CFUs per bottle (median: 16, range: 1 – 184). The inoculated blood culture bottles were incubated in the BACTEC 9050 automated blood culture system (Becton Dickinson) until positive. If positive blood culture bottles could not be tested by the BC-GN assay within 12 hours of blood culture positivity, positive bottles were refrigerated at 4°C for up to 48 hours. In some experiments, an equal volume from 2–3 positive blood culture bottles was mixed and the mixture was tested by the BC-GN assay.

### Clinical samples

Blood culture bottles from suspected sepsis patients were collected from December 2012 to June 2013 at the 801 bed National Center for Global Health and Medicine (NCGM), from February to June 2013 at 572 bed NCGM Kohnodai Hospital, and from March to June 2013 at 1423 bed Tokyo Women's Medical University (TWMU) hospital. Bactec plus/F (Becton Dickinson) and BacT/Alert FA (bioMérieux, Tokyo, Japan) blood culture bottles were used at NCGM and TWMU, respectively. Of 102 clinical samples, 79 and 23 were collected at NCGM and TWMU, respectively. Hospital departments and wards were not specified in the study. The bottles were incubated in the automated blood culture system until positive. Positive blood cultures showing Gram-negative bacteria were tested with the BC-GN assay. To select samples containing organisms listed in the BC-GN panel, positive blood cultures were stored at room temperature and tested within 5 days of positivity. The average of storage periods was 3.1±1.4. Only one positive blood culture per patient was included in the study.

### Identification of bacterial species and detection of resistance genes

Bacterial isolates were phenotypically identified using the MicroScan WalkAway™ system (Siemens Healthcare Diagnostics, Tokyo, Japan). Isolates generating identification discrepancies between the MicroScan WalkAway system and the BC-GN assay were analyzed by 16S ribosomal RNA (rRNA) sequencing [21]. DNA sequences were determined using an ABI PRISM3130 sequencer (Applied Biosystems, Foster City, CA). The sequence similarity was determined using the EzTaxon server (http://eztaxon-e.ezbiocloud.net/ezt_identify). The presence of 9 genes listed in the BC-GN panel was examined using PCR in simulated and clinical samples tested, regardless of the results with the BC-GN assay. The primers for PCR were shown in Table 1. The DNA sequences of the drug-resistant genes were determined when isolates generated discrepant results between the BC-GN assay and PCR.

**Table 1:** Primers used in this study.

Detection for antibiotic resistance genes		16S rRNA gene sequencing	
Primers	Sequence(5′-3′)	Primers	Sequence(5′-3′)
CTX-M-F	CGTTGTAAAACGACGGCCAGTGAA	5F	TTGGAGAGTTTGATCCTGGCTC
	TGTGCAGYACCAGTAARGTKATGGC	341F	CTACGGGAGGCAGCAGTGGG
CTX-M-R	TGGGTRAARTARGTSACCAGAAYCAGCGG	810R	GCGTGGACTTCCAGGGTATCT
IMP-F	GGAATAGAGTGGCTTAAYTCTC	1194R	ACGTCATCCCCACCTTCCTC
IMP-R	GGTTTAAYAAAACAACCACC	1485R	TACGGTTACCTTGTTACGAC
KPC-F	CGTCTAGTTCTGCTGTCTTG		
KPC-R	CTTGTCATCCTTGTTAGGCG		
NDM-F	GGTTTGGCGATCTGGTTTTC		
NDM-R	CGGAATGGCTCATCACGATC		
OXA-23like-F	GATCGGATTGGAGAACCAGA		
OXA-23like-R	ATTTCTGACCGCATTTCCAT		
OXA-24/40like-F	GGTTAGTTGGCCCCCTTAAA		
OXA-24/40like-R	AGTTGAGCGAAAAGGGGATT		
OXA48-F	GCGTGGTTAAGGATGAACAC		
OXA48-R	CATCAAGTTCAACCCAACCG		
OXA-58like-F	CCCCTCTGCGCTCTACATAC		
OXA-58like-R	AAGTATTGGGGCTTGTGCTG		
VIM-F	GATGGTGTTTGGTCGCATA		
VIM-R	CGAATGCGCAGCACCAG		

### Minimum inhibitory concentrations of antibiotics

Minimum inhibitory concentrations (MICs) of amikacin (AMK), ampicillin (ABPC), amoxicillin/clavulanate (AMPC/CVA), aztreonam (AZT), cefazolin (CEZ), cefepime (CFPM), cefmetazole (CMZ), cefotaxime (CTX), cefotiam (CTM), ceftazidime (CAZ), ciprofloxacin (CPFX), colistin, gentamicin (GM), imipenem (IPM), levofloxacin (LVFX), minocycline (MINO), meropenem (MEMP), piperacillin (PIPC), piperacillin-tazobactam (PIPC/TAZ), sulfamethoxazole-trimethoprim (ST), and tigecylcine (TGC) were determined by the Microscan Walkaway and/or the broth microdilution method, according to the guidelines of the Clinical and Laboratory Standards Institute (CLSI) [22]. Values of MICs at which 50% and 90% of *E. coli* isolates from clinical samples were inhibited (MIC_50_ and MIC_90_, respectively) were determined in β-lactams.

### Verigene System

The Verigene BC-GN assay detects 9 bacterial species and 9 drug resistance genes (Table 2). The Verigene System consists of the Verigene Reader and the Processor SP. Following loading of the extraction tray, utility tray and test cartridge into the Verigene Processor SP, 700 μl of positive blood culture was added to the extraction tray sample well. The Verigene Processor SP extracted nucleic acids from positive blood culture media. The extracted nucleic acids were automatically transferred to the test cartridge and hybridized to synthetic specific oligonucleotides attached to the microarray slide. The nucleic acids bound on the microarray slide were further hybridized to the second specific oligonucleotides with gold nanoparticles. After 2 hours, the microarray slide was manually removed and inserted into the Verigene Reader for analysis. The Verigene system contains internal controls, including negative controls, hybridization controls, and extraction controls. When any internal control do not generate correct results, or target signals were not adequately higher than the negative control signals, the Verigene Reader reported “No Call” meaning a technical error. When the Verigene Reader reported “No Call”, the BC-GN assay was repeated until results other than “No Call” were generated. Once the result was obtained, the BC-GN assay was not repeated.

**Table 2:** Bacterial species and Antimicrobial resistance genes identified by the BC-GN assay.

Gram-negative species
*Acinetobacter* spp.
*Citrobacter* spp.
*Enterobacter* spp.
*E. coli*
*K. oxytoca*
*K. pneumoniae*
*Proteus* spp.
*P. aeruginosa*
*S. marcescens*

Antimicrobial resistance genes
*bla*_CTX-M_
*bla*_IMP_
*bla*_KPC_
*bla*_NDM_
*bla*_OXA-23_
*bla*_OXA-24/40_
*bla*_OXA-48_
*bla*_OXA-58_
*bla*_VIM_

### Statistical analysis

The concordance rate between the BC-GN assay and standard laboratory methods was examined, and the 95% confidence interval of the rate was calculated with the R Software (http://www.r-project.org/).

### Ethical considerations

The study protocol was carefully reviewed and approved by the ethics committee of the National Center for Global Health and Medicine (No. 1268) and Tokyo Women's Medical University hospital (No. 2740-R), respectively. Individual informed consent was waived by the ethics committee listed above because this study used currently existing sample collected during the course of routine medical care and did not pose any additional risks to the patients.

## Results

### Bacterial identification of simulated samples

Of the 397 positive blood culture samples in the study, 295 were simulated samples prepared by inoculating an isolate of one of the Gram-negative bacterial species listed in Table 2. All the samples became culture-positive. Of the 295 samples, 289 generated a result of the BC-GN assay on the first attempt (98.1%) (data not shown). The remaining 6 generated “No Call ” results meaning technical errors. When retested, all the 6 samples generated results (data not shown). The concordance rate of the 295 simulated samples between the BC-GN assay and the MicroScan WalkAway system for bacterial identification were as follows: *Acinetobacter* spp., *Citrobacter* spp., *Enterobacter* spp., *E. coli*, *K. oxytoca*, *Proteus* spp., and *P. aeruginosa*: 100%; *K. pneumoniae*: 88.2%; and *S. marcescens*: 81.3% (left columns in Table 3).

**Table 3:** Identification of bacterial isolates in blood culture samples with the BC-GN assay.

	Simulated samples^b^ (n = 295)				Clinical samples^c^ (n = 102)			Total (n = 397)
Bacterial species^a^	Inocultaed isolates	Correctly identified isolates^d^ (%)	Not detected isolates^e^ (%)	Incorrectly identified isolates^f^ (%)	Identified species^g^	Correctly identified isolates^d^ (%)	Not detected isolates^e^ (%)	Concordance rate^h^ (95% CI^i^)
*Acinetobacter* spp.	37	37 (100%)	0	0	2	2 (100%)	0	100% (91.0–100)
*Citrobacter* spp.	6	6 (100%)	0	0	1	1 (100%)	0	100% (59.0–100)
*Enterobacter* spp.	18	18 (100%)	0	0	12	11 (91.7%)	1 (8.3%)	96.7% (82.8–99.9)
*E. coli*	36	36 (100%)	0	0	51	51 (100%)	0	100% (95.8–100)
*K. oxytoca*	6	6 (100%)	0	0	7	7 (100%)	0	100% (75.3–100)
*K. pneumoniae*	51	45 (88.2%)	5 (9.8%)	1^j^ (2.0%)	21	17 (81.0%)	4 (19%)	86.1% (75.9–93.1)
*Proteus* spp.	9	9 (100%)	0	0	2	2 (100%)	0	100% (91.0–100)
*P. aeruginosa*	116	116 (100%)	0	0	9	8 (88.9%)	1 (11.1%)	99.2% (95.6–99.9)
*S. marcescens*	16	13 (81.3%)	3 (18.7%)	0	5	5 (100%)	0	85.7% (63.7–97.0)
Total	295	286 (96.9%)	8 (2.7%)	1 (0.3%)	110	104 (94.5%)	6 (5.5%)	96.3% (94.0–97.9)

a Bacterial species inoculated into blood culture bottles or bacterial species identified in clinical samples of blood culture bottles.

b Blood culture samples into which bacterial isolates were inoculated.

c Clinical blood culture samples obatined from sepsis patients.

d Numbers of isolates which were correctly identified with the BC-GN assay.

e Numbers of isolates which were not detected with the BC-GN assay.

f Numbers of isolates which were incorrectly identified with theBC-GN assay.

g Numbers of bacterial species which were identified in clinical samples with the conventional methods. Some of the samples contained 2 or 3 bacterial species.

h Concordance rate between the BC-GN assay and the conventional methods for bacterial identification.

i CI indicates confidence interval

j A sample inoculated with K. pneumoniae was identified as Enterobacter spp. with the assay.

Of 51 samples inoculated with stored *K. pneumoniae* isolates (left columns in Table 3), 45 were detected with the BC-GN assay, but the remaining 6 were not correctly detected. Of these 6 samples, 5 were reported as “Not detected”, and 1 was reported as *Enterobacter* spp. The MicroScan WalkAway system reported these 6 samples as *K. pneumoniae*. The 16S rRNA sequences of these samples showed more than 99.3% similarity to both *K. pneumoniae* and *K. variicola*. Of 16 samples inoculated with stored *S*. *marcescens* (left columns in Table 3), 3 were not detected with the BC-GN assay. The MicroScan WalkAway system reported these 3 samples as *S*. *marcescens*. The 16S rRNA sequences of these samples showed more than 99.5% similarity to both *S*. *marcescens* and *Serratia nematodiphila*. Their biochemical profile based on carbohydrates utilization corresponded to those of *S*. *marcescens* [23].

The BC-GN assay was tested on blood culture bottles inoculated simultaneously with 2 or 3 clinical isolates. The following combinations of bacteria species were tested: *A. baumannii* (harboring *bla*_OXA-23_) and *K*. *pneumoniae* (*bla*_CTX-M_ and *bla*_NDM_); *A. baumannii* (*bla*_OXA-23_) and MRSA; *C. freundii*, *K*. *pneumoniae* (*bla*_KPC_) and *P. mirabilis*; *C. freundii* (*bla*_KPC_), *E. faecalis* and MSSA; *E. cloacae* (*bla*_IMP_) and *S*. *marcescens*; *E. faecalis* and *E. coli* (*bla*_CTX-M_); *E. coli* (*bla*_CTX-M_) and *P*. *aeruginosa* (*bla*_VIM_); *P*. *aeruginosa* (*bla*_VIM_) and MRSE. All Gram-negative bacterial isolates were correctly detected by the BC-GN assay regardless of the presence of other bacterial species, including Gram-positive strains. The BC-GN assay also detected all drug resistance genes under these conditions (data not shown). The BC-GN assay was also tested on 2 blood culture bottles inoculated with *M. morganii* and *S. maltophilia*, respectively, which are Gram-negative pathogens but are not among the targets of the BC-GN assay. As expected, the BC-GN assay did not detect these species (data not shown).

### Bacterial identification of clinical samples

A total of 102 blood culture-positive samples obtained from sepsis patients, and 101 of them generated a result of the BC-GN assay on the first attempt (99.0%) (data not shown). From the 102 blood culture-positive samples, a total of 110 Gram-negative bacterial species were isolated (middle columns in Table 3). The concordance rate of 110 bacterial species between the BC-GN assay and the MicroScan WalkAway system for bacterial identification were 94.5% (NCGM: 82/86; TWMU: 22/24), and the rate of each bacterial species were as follows: *Acinetobacter* spp., *Citrobacter* spp., *E. coli*, *K. oxytoca*, *Proteus* spp., and *S. marcescens*: 100%; *Enterobacter* spp.: 91.7%; *K. pneumoniae*: 81.0%; and *P. aeruginosa*: 88.9% (middle columns in Table 3). Of all the clinical samples, 8 were obtained from anaerobic bottles, and all the 8 results of the BC-GN assay agreed with those of the MicroScan WalkAway system (data not shown).

Of the 110 bacterial isolates, 6 isolates (5.5%) were not detected with the BC-GN assay (middle columns in Table 3). Of these 6 isolates, the MicroScan WalkAway system reported 1 isolate as *Enterobacter* spp., 4 isolates as *K*. *pneumoniae* and 1 isolate as *P. aeruginosa* (middle columns in Table 3). The sample reported as *Enterobacter* spp. by the MicroScan WalkAway system was reported as “No Call” with the BC-GN assay on two tries. The 16S rRNA sequence of the isolate reported as *Enterobacter* spp. by the MicroScan WalkAway system showed more than 99.9% similarity to *Enterobacter agglomerance*. Of the 4 samples reported as *K*. *pneumoniae* by the MicroScan WalkAway system and not detected by the BC-GN assay, 2 were from polymicrobial bacteremia cases: *E. coli* and *K. pneumoniae* was isolated from one positive bottle, and *K. pneumoniae* and *P. aeruginosa* was isolated from another. The 16S rRNA sequences of the 4 isolates reported as *K*. *pneumoniae* by the MicroScan WalkAway system showed more than 99.2% similarity to both *K. pneumoniae* and *K. variicola*. One *P*. *aeruginosa* sample not detected with the BC-GN was from a polymicrobial bacteremia case of *K. pneumoniae* and *P. aeruginosa*. The 16S rRNA sequence of the isolate reported as *P*. *aeruginosa* by the MicroScan WalkAway system showed more than 99.9% similarity to *P*. *aeruginosa*.

Seven of the 102 clinical samples were polymicrobial. In 4 of the 7 samples, the BC-GN assay detected all of the multiple bacterial species, even in a sample containing 3 different Gram-negative species. In a sample containing *Enterococcus casseliflavus* and *E. coli*, the BC-GN assay detected only *E. coli* but not *E*. *casseliflavus*, since it detects only Gram-negative bacteria but not Gram-positive ones. In the remaining 3 polymicrobial positive blood culture samples, the BC-GN assay did not detect one of the multiple pathogens (*K. pneumoniae* in 2 samples and *P. aeruginosa* in 1 samples). The bacterial identification of the samples not detected by the BC-GN assay was described above.

### Identification of drug resistance genes

With respect to drug resistant genes, the BC-GN assay reported that, of the 295 simulated samples tested, 184 were positive for one of the resistance gene targets detected by the BC-GN assay and 18 were positive for two of the targets. These results agreed with those of PCR and/or DNA sequencing. As shown on left columns in Table 4, 42 *bla*_CTX-M_ (2 *E. cloacae*, 19 *E. coli*, 2 *E. hormaechei*, 18 *K. pneumoniae*, and 1 *P. mirabilis* isolates), 119 *bla*_IMP_ (1 *A. calcoaceticus*, 1 *A. nosocomialis*, 1 *A. pittii*, 9 *E. cloacae*, 4 *K. pneumoniae*, and 103 *P. aeruginosa*), 8 *bla*_KPC_ (2 *C. freundii*, 5 *K. pneumoniae*, and 1 *S. marcescens*), 16 *bla*_NDM_ (2 *A. baumannii*, 1 *E. hormaechei*, 1 *E. coli*, 11 *K. pneumoniae*, and 1 *P. aeruginosa*), 24 *bla*_OXA-23_ (24 *A. baumannii*), 1 *bla*_OXA-24/40_ (1 *A. baumannii*), 1 *bla*_OXA-48_ (1 *K. pneumoniae*), 3 *bla*_OXA-58_ (2 *A. baumannii* and 1 *A. pittii*), and 6 *bla*_VIM_ (6 *P. aeruginosa*) were detected by the BC-GN assay.

**Table 4:** Identification of resistance genes in blood culture samples with the BC-GN assay.

Resistance genes^a^	Simulated samples^b^ (n = 295)			Clinical samples^c^ (n = 102)			Total (n = 397)
	Positive genes for PCR/DNAsequences	Correctly identified genes^d^ (%)	Not detected genes^e^	Positive genes for PCR/DNAsequences	Correctly identified genes^d^ (%)	Not detected genes^e^	Concordance rate^f^ (95% CI^g^)
*bla*_CTX-M_	42	42 (100%)	0	12	12 (100%)	0	100% (93.3–100)
*bla*_IMP_	119	119 (100%)	0	0	0	0	100% (96.9–100)
*bla*_KPC_	8	8 (100%)	0	0	0	0	100% (63.1–100)
*bla*_NDM_	16	16 (100%)	0	0	0	0	100% (79.4–100)
*bla*_OXA-23_	24	24 (100%)	0	0	0	0	100% (85.8–100)
*bla*_OXA-24/40_	1	1 (100%)	0	0	0	0	100% (2.5–100)
*bla*_OXA-48_	1	1 (100%)	0	0	0	0	100% (2.5–100)
*bla*_OXA-58_	3	3 (100%)	0	1	1 (100%)	0	100% (39.8–100)
*bla*_VIM_	6	6 (100%)	0	0	0	0	100% (54.1–100)
Total	220	220 (100%)	0	13	13 (100%)	0	100% (98.4–100)

a Resistance genes which were harbored by bacterial species inoculated into blood culture bottles or isolated from sepsis patients.

b, c See footnotes b and c in Table 3.

d Numbers of genes which were correctly identified with the BC-GN assay.

e Numbers of genes which were not detected with the BC-GN assay.

f Concordance rate between the BC-GN assay and the conventional methods for resistance-genes identification.

g CI indicates confidence interval

Of the 102 clinical samples tested, 13 were positive for one of the 9 drug resistance genes. As shown on middle columns in Table 4, 12 *bla*_CTX-M_ (2 *E. cloacae*, 8 *E. coli*, and 2 *K. pneumoniae*), and 1 *bla*_OXA-58_ (1 *A. baumannii*) were detected by the BC-GN assay. These results were completely agreed with those of our laboratory methods.

MICs of *E. coli* isolates from patients harboring or not harboring *bla*_CTX-M_ were determined (Table 5). The *E. coli* isolates harboring *bla*_CTX-M_ showed higher MIC_50_ and MIC_90_ of β-lactams tested than the isolates not harboring *bla*_CTX-M_. MICs of IMP were ≤1 μg/ml in *E. coli* isolates harboring or not harboring *bla*_CTX-M_. An isolate of *Acinetobacter* spp. harboring *bla*_OXA-58_ was susceptible to all β-lactams tested (Table S1). MICs of all clinical isolates were shown in Table S1, 2.

**Table 5:** Drug susceptibility of β-lactams of E. coli harboring or not harboring.

			β-lactams										
		Number of isolates	PIPC			CAZ			CTX			IMP	
			MIC_50_	MIC_90_		MIC_50_	MIC_90_		MIC_50_	MIC_90_		MIC_50_	MIC_90_
			(μg/ml)	(μg/ml)		(μg/ml)	(μg/ml)		(μg/ml)	(μg/ml)		(μg/ml)	(μg/ml)
*E. coli* (*bla*_CTX-M_ -positive)		8	>512	>512		8	>16		>32	>32		≤1	≤1
*E. coli* (*bla*_CTX-M_ -negative)		43	≤8	>64		≤1	≤1		≤8	≤8		≤1	≤1

## Discussion

We determined the overall concordance rate, 96.3%, between the BC-GN assay and standard laboratory methods (Table 3). All bacterial species tested except for *K. pneumoniae* and *S. marcescens* showed the concordance rate of over 95%. In addition to the good performance, the BC-GN assay can be completed within 2 h with less than 5 min of hands-on time, enabling same day analysis and starting on appropriate treatment. The concordance rate of the BC-GN assay was comparable to that of the BC-GP assay. However, the BC-GN assay will need to be evaluated using various blood culture systems, since the BC-GP assay have already evaluated as well [12]–[19].

The BC-GN assay did not accurately identify a total of 15 isolates from 9 simulated samples and 6 clinical samples (Table 3). Of the 15 isolates, 10, 3, 1 and 1 were reported as *K*. *pneumoniae*, *S. marcescens*, *Enterobacter* spp., *P. aeruginosa* by the MicroScan WalkAway system, respectively. Relatively higher rates of misidentification in *K*. *pneumoniae* and *S. marcescens* isolates were likely to be due to the probes used in the BC-GN assay. These probes will be improved in the further study. The concordance rates of Gram-positive bacteria were comparable in the BC-GP assay [12]–[19]. The lower accuracy results of *K*. *pneumoniae* were shown in both the spiked and clinical observations. The 16S rRNA sequence revealed that the 10 misidentified isolates shared over 99% similarity to both *K*. *pneumoniae* and *K. variicola*. Previous studies have reported that routine methods may identify *K. variicola* as *K. pneumoniae* [24]. Further studies are required to clarify the clinical significance of *K. variicola* in sepsis patients. As described above, three samples containing *S. marcescens* were not detected with the BC-GN assay, which were observed only in the simulated samples. It is unclear whether clinical samples containing *S. marcescens* were not detected accurately with the BC-GN assay, since the number of samples tested was low.

As for the detection of polymicrobial samples, the BC-GN assay performed well in the simulated samples, but not in the clinical samples. All isolates were not detected completely in 3 of 7 clinical samples containing polymicrobial organisms. Although fungal species like *Candida albicans* were not tested, these results can have significant clinical implications. Rapid molecular-based assays including the BC-GP assay and the FilmArray blood culture identification assay showed relatively low sensitivity in polymicrobial infections [14], [25].

We evaluated the performance of the BC-GN assay in detecting various drug resistance genes using simulated samples. The BC-GN assay correctly detected all the drug resistance genes tested, even when multiple drug resistance genes were present in a sample. In addition, MICs of *E. coli* isolates from clinical samples indicated that the presence of *bla*_CTX-M_ was related to drug susceptibility profiles with high MICs of PIPC, CAZ, and CTX, not IPM. These results suggest that the BC-GN assay is useful for earlier administration of appropriate antibiotics and de-escalation. Whereas, no genotypic-phenotypic correlation was found in the *Acinetobacter* spp. isolate harboring *bla*_OXA-58_. In that case, the BC-GN assay may lead to inappropriate use of antibiotic, and de-escalation is needed after conventional drug susceptibility testing.

Although *bla*_CTX-M_ is the most prevalent among genes of extended-spectrum β-lactamases (ESBLs)[26], [27], the BC-GN assay cannot detect other ESBLs genes like *bla*_TEM_ and *bla*_SHV_, and there remains concern of bacteria harboring drug resistance genes which were not detected by the assay. More than 200 *bla*_TEM_ and more than 100 *bla*_SHV_ genes are currently known in the Lahey clinic database [28]. Thus, it remains unfeasible to detect these variants in a rapid and simple manner. The emergence of various β-lactam resistance genes, including carbapenemases and ESBLs, have been extensively reported [6]. The timely direct detection of resistance genes from positive blood cultures will be very useful in clinical situations, since multidrug-resistant bacteria and polymicrobial infections are important causes of failure in the treatment of sepsis. Because drug resistance genes will continue to evolve and spread, it is necessary to carefully monitor the spread of drug resistance genes.

Of the 10 most frequently isolated microorganisms in ICU-acquired bloodstream infection in Europe, 7 were Gram-negative pathogens targeted by the BC-GN assay [29]. Similar results were obtained from monomicrobial nosocomial bloodstream infections both in ICU and non-ICU wards in the USA [30]. The National Healthcare Safety Network at CDC reported that a significant proportion of central line-associated bloodstream infections are caused by drug-resistant Gram-negative pathogens in the USA [31]. Of the 6 major pathogens other than coagulase-negative staphylococci associated with nosocomial blood stream infections in Japan, 3 were Gram-negative pathogens, 2 were Gram-positive ones, and 1 was *Candida* spp. [32]. The BC-GN assay, which targets 9 Gram-negative bacterial species, will contribute to the diagnosis and management of patients with Gram-negative sepsis.

A number of new diagnostic methods have been developed for rapid detection of pathogens in positive blood cultures. A real-time PCR-based assay (LightCycler SeptiFast Test) can detect a number of Gram-negative and Gram-positive bacteria and fungi in a single assay, although it cannot detect drug resistance genes [8]. PNA-FISH can detect 3 Gram-negative pathogens, including *E. coli*, *K. pneumoniae*, and *P. aeruginosa*, but cannot detect drug resistance genes [9]. MALDI-TOF MS can detect bacterial isolates in blood culture, but cannot detect 2 or more bacterial species in polymicrobial samples or drug resistance gene products [33]. A DNA-based microarray platform, Probe-it sepsis assay, can detect various Gram-negative and Gram-positive pathogens in blood cultures. Nevertheless, it cannot detect drug resistance genes other than *mecA* [11].

To integrate the Verigene system into a laboratory workflow, Gram stain was needed after blood culture was positive to determine whether the BC-GN or BC-GP assay was used. The Verigene system cannot measure MICs of antibiotics, and thus should be used in combination with biochemical characteristics of bacteria obtained from conventional drug susceptibility testing.

This study has some limitations. Firstly, we could not fully evaluate the ability of the BC-GN assay to detect some drug resistance genes, such as *bla*_OXA-24/40_ and *bla*_OXA-48_. Secondly, to select samples containing organisms listed in the BC-GN panel, positive blood culture was stored for 3.1±1.4 days (maximum 5 days) before the BC-GN assay was done. The BC-GN assay should be done immediately after the culture becomes positive, although the storage of the culture in the study was unlikely to affect the assay results. A large-scale prospective clinical evaluation of the BC-GN assay is planned to determine the clinical impact of the assay including selection of antibiotics, length of stay, morbidity, and cost-effectiveness.

In conclusion, the Verigene system BC-GN assay accurately detects common Gram-negative bacterial isolates and their drug resistance genes from positive blood cultures in a rapid manner. The BC-GN assay demonstrated high sensitivity and specificity in the study, and it will generate significantly faster results compared to conventional methods in the medical settings. This new anitibioticroarray platform can be easily introduced into microbiological laboratories in various clinical settings. It will contribute to improved sepsis management as a result of earlier reporting of critical information leading to earlier administration of appropriate antibiotics and de-escalation.

## Supporting Information

Table S1 **MICs of antibiotics in clinical isolates harboring drug resistance genes.** It was measured using the broth microdilution method, according to the guidelines of the Clinical and Laboratory Standards Institute (CLSI).
(XLSX)

Table S2 **MICs of antibiotics in all clinical isolates determined by Microscan Walkaway.**
(XLSX)

## References

[1] Peleg AY, Hooper DC (2010) Hospital-acquired infections due to gram-negative bacteria. N Engl J Med 362: 1804–1813. 20463340 10.1056/NEJMra0904124 PMC3107499

[2] Shorr AF, Micek ST, Welch EC, Doherty JA, Reichley RM, et al (2011) Inappropriate antibiotic therapy in Gram-negative sepsis increases hospital length of stay. Crit Care Med 39: 46–51. 20890186 10.1097/CCM.0b013e3181fa41a7

[3] Gaieski DF, Mikkelsen ME, Band RA, Pines JM, Massone R, et al (2010) Impact of time to antibiotics on survival in patients with severe sepsis or septic shock in whom early goal-directed therapy was initiated in the emergency department. Crit Care Med 38: 1045–1053. 20048677 10.1097/CCM.0b013e3181cc4824

[4] Kumar A, Roberts D, Wood KE, Light B, Parrillo JE, et al (2006) Duration of hypotension before initiation of effective antimicrobial therapy is the critical determinant of survival in human septic shock. Crit Care Med 34: 1589–1596. 16625125 10.1097/01.CCM.0000217961.75225.E9

[5] Versalovic J, Carroll KC, Funke G, Jorgensen JH, Landry ML, et al.. (2011) Systems for Detection and Identification of Bacteria and yeasts. Manual of clinical microbiolgy. ASM Press, Washington, DC. pp. 15–26.

[6] Queenan AM, Bush K (2007) Carbapenemases: the versatile beta-lactamases. Clin Microbiol Rev 20: : 440–58, table of contents. 10.1128/CMR.00001-07 PMC1932750 17630334

[7] Liesenfeld O, Lehmann LE, Hunfeld K-P, Kost G (2014) Molecular Diagnosis of Sepsis: New aspects and recent developments. Eur J Microbiol Immunol: in press. 10.1556/EuJMI.4.2014.1.1 PMC3955828 24678402

[8] Mancini N, Clerici D, Diotti R, Perotti M, Ghidoli N, et al (2008) Molecular diagnosis of sepsis in neutropenic patients with haematological malignancies. J Med Microbiol 57: 601–604. 18436593 10.1099/jmm.0.47732-0

[9] Morgan M, Marlowe E, Della-Latta P, Salimnia H, Novak-Weekley S, et al (2010) Multicenter evaluation of a new shortened peptide nucleic acid fluorescence in situ hybridization procedure for species identification of select Gram-negative bacilli from blood cultures. J Clin Microbiol 48: 2268–2270. 20357212 10.1128/JCM.00166-10 PMC2884511

[10] Leggieri N, Rida A, François P, Schrenzel J (2010) Molecular diagnosis of bloodstream infections: planning to (physically) reach the bedside. Curr Opin Infect Dis 23: 311–319. 20592531 10.1097/QCO.0b013e32833bfc44

[11] Tissari P, Zumla A, Tarkka E, Mero S, Savolainen L, et al (2010) Accurate and rapid identification of bacterial species from positive blood cultures with a DNA-based microarray platform: an observational study. Lancet 375: 224–230. 20004964 10.1016/S0140-6736(09)61569-5

[12] Samuel LP, Tibbetts RJ, Agotesku A, Fey M, Hensley R, et al (2013) Evaluation of a microarray-based assay for rapid identification of Gram-positive organisms and resistance markers in positive blood cultures. J Clin Microbiol 51: 1188–1192. 23363838 10.1128/JCM.02982-12 PMC3666768

[13] Wojewoda CM, Sercia L, Navas M, Tuohy M, Wilson D, et al (2013) Evaluation of the Verigene Gram-positive blood culture nucleic acid test for rapid detection of bacteria and resistance determinants. J Clin Microbiol 51: 2072–2076. 23596240 10.1128/JCM.00831-13 PMC3697701

[14] Buchan BW, Ginocchio CC, Manii R, Cavagnolo R, Pancholi P, et al (2013) Multiplex identification of gram-positive bacteria and resistance determinants directly from positive blood culture broths: evaluation of an automated microarray-based nucleic acid test. PLoS Med 10: e1001478. 23843749 10.1371/journal.pmed.1001478 PMC3699453

[15] Sullivan KV, Turner NN, Roundtree SS, Young S, Brock-Haag CA, et al (2013) Rapid detection of Gram-positive organisms by use of the Verigene Gram-positive blood culture nucleic acid test and the BacT/Alert Pediatric FAN system in a multicenter pediatric evaluation. J Clin Microbiol 51: 3579–3584. 23966484 10.1128/JCM.01224-13 PMC3889736

[16] Alby K, Daniels LM, Weber DJ, Miller MB (2013) Development of a treatment algorithm for streptococci and enterococci from positive blood cultures identified with the Verigene Gram-positive blood culture assay. J Clin Microbiol 51: 3869–3871. 23985910 10.1128/JCM.01587-13 PMC3889785

[17] Beal SG, Ciurca J, Smith G, John J, Lee F, et al (2013) Evaluation of the nanosphere verigene gram-positive blood culture assay with the VersaTREK blood culture system and assessment of possible impact on selected patients. J Clin Microbiol 51: 3988–3992. 24048531 10.1128/JCM.01889-13 PMC3838064

[18] Sango A, McCarter YS, Johnson D, Ferreira J, Guzman N, et al (2013) Stewardship approach for optimizing antimicrobial therapy through use of a rapid microarray assay on blood cultures positive for Enterococcus species. J Clin Microbiol 51: 4008–4011. 24068006 10.1128/JCM.01951-13 PMC3838051

[19] Mestas J, Polanco CM, Felsenstein S, Dien Bard J (2014) Performance of the Verigene Gram-positive blood culture assay for direct detection of Gram-positive organisms and resistance markers in a pediatric hospital. J Clin Microbiol 52: 283–287. 24131696 10.1128/JCM.02322-13 PMC3911431

[20] Kouyama Y, Harada S, Ishii Y, Saga T, Yoshizumi A, et al (2012) Molecular characterization of carbapenem-non-susceptible Acinetobacter spp. in Japan: predominance of multidrug-resistant Acinetobacter baumannii clonal complex 92 and IMP-type metallo-β-lactamase-producing non-baumannii Acinetobacter species. J Infect Chemother 18: 522–528. 22327516 10.1007/s10156-012-0374-y

[21] Sontakke S, Cadenas MB, Maggi RG, Diniz PPVP, Breitschwerdt EB (2009) Use of broad range16S rDNA PCR in clinical microbiology. J Microbiol Methods 76: 217–225. 19046999 10.1016/j.mimet.2008.11.002

[22] Clinical Laboratory Standards Institute (2009) Methods for dilution antimicrobial susceptibility tests for bacteria that grow aerobically; approved standard M07-A7. 8th ed. Wayne, PA.

[23] Zhang CX, Yang SY, Xu MX, Sun J, Liu H, et al (2009) Serratia nematodiphila sp. nov., associated symbiotically with the entomopathogenic nematode Heterorhabditidoides chongmingensis (Rhabditida: Rhabditidae). Int J Syst Evol Microbiol 59: 1603–1608. 19578149 10.1099/ijs.0.65718-0

[24] Seki M, Gotoh K, Nakamura S, Akeda Y, Yoshii T, et al (2013) Fatal sepsis caused by an unusual Klebsiella species that was misidentified by an automated identification system. J Med Microbiol 62: 801–803. 23449877 10.1099/jmm.0.051334-0

[25] Altun O, Almuhayawi M, Ullberg M, Ozenci V (2013) Clinical evaluation of the FilmArray blood culture identification panel in identification of bacteria and yeasts from positive blood culture bottles. J Clin Microbiol 51: 4130–4136. 24088863 10.1128/JCM.01835-13 PMC3838040

[26] Fujita S, Yosizaki K, Ogushi T, Uechi K, Takemori Y, et al (2011) Rapid identification of gram-negative bacteria with and without CTX-M extended-spectrum β-lactamase from positive blood culture bottles by PCR followed by microchip gel electrophoresis. J Clin Microbiol 49: 1483–1488. 21289149 10.1128/JCM.01976-10 PMC3122828

[27] Cantón R, Coque TM (2006) The CTX-M beta-lactamase pandemic. Curr Opin Microbiol 9: 466–475. 16942899 10.1016/j.mib.2006.08.011

[28] Bush K, Jacoby GA (2010) Updated functional classification of beta-lactamases. Antimicrob Agents Chemother 54: 969–976. 19995920 10.1128/AAC.01009-09 PMC2825993

[29] European Centre for disease prevention and Control (ECDC) (2012) Healthcare-associated infections. Annual epidemiological report Reporting on 2010 surveillance data and 2011 epidemic intelligence data. p. 212.

[30] Wisplinghoff H, Bischoff T, Tallent SM, Seifert H, Wenzel RP, et al (2004) Nosocomial bloodstream infections in US hospitals: analysis of 24,179 cases from a prospective nationwide surveillance study. Clin Infect Dis 39: 309–317. 15306996 10.1086/421946

[31] Sievert DM, Ricks P, Edwards JR, Schneider A, Patel J, et al (2013) Antimicrobial-resistant pathogens associated with healthcare-associated infections: summary of data reported to the National Healthcare Safety Network at the Centers for Disease Control and Prevention, 2009-2010. Infect Control Hosp Epidemiol 34: 1–14. 23221186 10.1086/668770

[32] Nagao M (2012) A multicentre analysis of epidemiology of the nosocomial bloodstream infections in Japanese university hospitals. Clin Microbiol Infect: Article first published online. 10.1111/1469-0691.12083 23176224

[33] La Scola B, Raoult D (2009) Direct identification of bacteria in positive blood culture bottles by matrix-assisted laser desorption ionisation time-of-flight mass spectrometry. PLoS One 4: e8041. 19946369 10.1371/journal.pone.0008041 PMC2777307

